# Astrogliosis Induced by Brain Injury Is Regulated by Sema4B Phosphorylation^1^,^2^,^3^

**DOI:** 10.1523/ENEURO.0078-14.2015

**Published:** 2015-05-25

**Authors:** Liat Ben-Gigi, Sahar Sweetat, Elazar Besser, Yakov Fellig, Thorsten Wiederhold, Roberto D. Polakiewicz, Oded Behar

**Affiliations:** 1 Department of Developmental Biology and Cancer Research, Institute of Medical Research Israel-Canada (IMRIC)], Faculty of Medicine, The Hebrew University, Jerusalem 91120, Israel; 2 Neuropathology Unit, Department of Pathology, Hadassah Medical Center, The Hebrew University, Jerusalem 91120, Israel; 3 Cell Signaling Technology, Inc., Danvers, Massachusetts 01923

**Keywords:** astrogliosis, CNS injury, Sema4B

## Abstract

Injury to the CNS induces astrogliosis, an astrocyte-mediated response that has both beneficial and detrimental impacts on surrounding neural and non-neural cells. The precise signaling events underlying astrogliosis are not fully characterized. Here, we show that astrocyte activation was altered and proliferation was reduced in Semaphorin 4B (Sema4B)-deficient mice following injury. Proliferation of cultured Sema4B^−/−^ astrocytes was also significantly reduced. In contrast to its expected role as a ligand, the Sema4B ectodomain was not able to rescue Sema4B^−/−^ astrocyte proliferation but instead acted as an antagonist against Sema4B^+/−^ astrocytes. Furthermore, the effects of Sema4B on astrocyte proliferation were dependent on phosphorylation of the intracellular domain at Ser825. Our results suggest that Sema4B functions as an astrocyte receptor, defining a novel signaling pathway that regulates astrogliosis after CNS injury.

## Abstract (precis)

Astrocyte activation plays a critical role in response to CNS trauma. Following CNS injury, astrogliosis has the beneficial effect of restricting tissue damage, but it also limits neuronal regeneration.

## Significance Statement

Astrocyte activation plays a critical role in response to CNS trauma. Following CNS injury, astrogliosis has the beneficial effect of restricting tissue damage, but it also limits neuronal regeneration. That modulation of astrogliosis may improve neuronal regeneration is a widely held view. However, the cellular and molecular mechanisms underlying astrogliosis are not fully characterized.

Here, we identify the involvement of an unexpected protein, Sema4B, a transmembrane member of the semaphorin family of proteins, in modulating astrocyte activation and proliferation in the aftermath of CNS injury. Although to date Sema4B has been shown to function as a ligand, our present results in astrocytes are more consistent with its function as a receptor or as a signaling molecule.

## Introduction

Brain damage as a result of stroke or head trauma is one of the leading causes of disability and death in humans. Brain trauma activates astrocytes in a process called reactive astrogliosis, which initiates changes in molecular expression and morphology, and, in severe cases, scar formation (Sofroniew and Vinters, 2010). Although astrogliosis typically occurs in response to many types of insults, recent studies have demonstrated clear differences in gene expression profiles between astrocytes activated by inflammation and those activated by ischemic stroke (Zamanian et al., 2012). The overall effect of reactive astrocytes on injury outcome is not entirely clear, although accumulating evidence indicates that it may have both positive and negative consequences. Experimental ablation of astrocytes slows recovery from CNS injury, implying that the presence of reactive astrocytes is crucial under such conditions (Bush et al., 1999; Faulkner et al., 2004). However, the effect of astrogliosis on postinjury events may differ according to the specific signaling molecules involved. For example, deletion of STAT3 in astrocytes results in increased neuronal death and reduced recovery following CNS injury (Okada et al., 2006; Herrmann et al., 2008), whereas inhibition of nuclear factor-κB in astrocytes improves recovery (Brambilla et al., 2005). It thus appears that distinct signaling cascades specifically influence astroglial function to determine the outcome of brain pathology and the degree of neurological damage.

Upon injury, the astroglial response is evoked by several changes occurring in the CNS parenchyma. A variety of signals are produced, including cytokines and growth factors such as Sonic Hedgehog and epidermal growth factor (EGF; Amankulor et al., 2009; Codeluppi et al., 2009). Although astrogliosis can potentially be triggered by any of these signals, the glial response itself is highly diverse, and may thus evoke additional signaling receptors and ligands. Since postinjury astrocytic borders and cell proliferation must be coordinated among the migrating and proliferating astrocytes, transmembrane ligand−receptor sets may serve as efficient mediators for communicating and coordinating this complex response. Members of the semaphorin family are good candidates for such functions. Some members of this gene family commonly act as ligands that bind directly to plexins (Pasterkamp, 2012). However, they may also operate in a reverse-signaling mechanism as receptors, as shown for Semaphorin 6D (Sema6D)/PlexinA1 (Toyofuku et al., 2004). Semaphorins are upregulated after injury, and numerous studies have shown that they regulate both cell proliferation and cell migration, making them intriguing candidates for postinjury astrocyte regulation (Pasterkamp and Verhaagen, 2006).

Because of its expression in astrocytes, Sema4B, a type 4 transmembrane semaphorin, is of particular interest in this context (Cahoy et al., 2008; Maier et al., 2011). Here, we examined the role of Sema4B in astrogliosis following brain injury.

## Materials and Methods

### Antibodies, growth factors, and materials

EGF was obtained from Peprotech. Anti-Iba1 was obtained from Wako. Anti-S100β was obtained from Abcam and from DAKO, anti-β-galactosidase (β-gal) was obtained from Abcam. Anti-glial fibrillary acid protein (GFAP), anti-phospho (p)-Sema4B, anti-Sema4B, anti-vimentin, anti-tubulin α/β, anti-p-ERK1/2, anti-Ki-67, anti-myc, and anti-NeuN were purchased from Cell Signaling Technology. Anti-bromodeoxyuridine (BrdU; G3G4, developed by S.J. Kaufman, was obtained from the Developmental Studies Hybridoma Bank, which was developed under the auspices of the National Institute of Child Health and Human Development and maintained at the University of Iowa, Department of Biological Sciences, Iowa City, IA). BrdU flow kit was purchased from (BD Biosciences). Secondary antibodies were obtained from Jackson ImmunoResearch. All other reagents were purchased from Sigma.

### Animals and surgical procedures

Sema4B^+/−^ mutant mice were purchased from the Mutant Mouse Regional Resource Center. This mouse line was generated as a result of targeted trap alleles. In this mouse line, Sema4B is retained within the intracellular compartment (Friedel et al., 2005). A heterozygous breeding strategy was adopted in order to obtain both wild-type, heterozygous (Sema4B^+/−^) and mutant (Sema4B^−/−^) mice. Both sexes were used in the experiments. All animal procedures were performed according to the regulations of the authors’ university animal care committee.

All cortical injury experiments were performed on mice that were 7 − 8 weeks old. Genotype was determined by PCR analysis of genomic DNA isolated from tail clippings of 3-week-old mice. The presence of the wild-type Sema4B allele was established using primer 1 (5'-AGACATGGTGCTGGAGAGGT-3') with primer 2 (5'-TGTGTTTGGTTGGATCTGGA-3'). The mutant allele of Sema4B^−/−^ was verified with primer 3 (5'-TGCACATGCTTTACGTGTG-3') and primer 4 (5'-TGCCGCGTGTCGTGTTGCAC-3').

For the injury experiments, mice were anesthetized with a ketamine/xylazine solution (50 mg/kg ketamine/7.5 mg/kg xylazine in 0.9% NaCl solution). A sterile needle was inserted vertically into the right cerebral hemisphere, reaching the skull surface at a depth of 5 mm. The needle was inserted through the cranium 2 mm caudal to the bregma and 1 mm lateral to the midline. The skin incision was closed with sutures.

### Assays

#### Immunoblots

Astrocyte cultures (or tissue biopsy samples of the injury site) were harvested in lysis buffer (1% NP40, 0.5% sodium deoxycholate, 0.1% SDS, 150 mm NaCl, 10 mm buffered phosphate, pH 7.2, 2 mm EDTA, 50 mm NaF, 0.2 mm orthovanadate, and protease inhibitor cocktail). Cells were collected with a cell scraper, passed six times through a pipette tip, vortexed, and incubated on ice for 15 min. The lysates were then centrifuged at 20,000 × *g* for 15 min, and pellets were discarded. Protein concentration of each sample was determined using Bradford Reagent (Sigma). Samples containing 20 mg of protein were boiled in 1× SDS sample buffer, separated by SDS (10%)-PAGE and blotted onto PVDF membranes (Millipore). The membranes were incubated in 5% fat-free milk in TBST (10 mm Tris-HCL, pH 7.4, 150 mm NaCl, and 0.1% Tween 20) for 1 h and then 5% BSA in TBST containing various dilutions of primary antibodies for 18 h at 4°C. The membranes were washed three times with TBST for 5 min each before and after incubation with secondary antibody. The proteins were detected with an appropriate secondary antibody (for 1 h at room temperature) coupled to horseradish peroxidase-conjugated goat anti-rabbit or anti-mouse antibody and visualized by chemiluminescence according to the manufacturer instructions (Pierce).

#### Bromodeoxyuridine injection

BrdU (Sigma) was dissolved in 0.9% NaCl at a concentration of 10 µg/µl. For labeling of dividing cells, the injured mice received injections of BrdU (50 mg/kg body weight, i.p.) on days 3, 5, and 7 after injury. Mice were killed 3 h after the final injection, 7 d postinjury.

#### Quantitative PCR analysis

Total RNA (500 ng) in a total volume of 20 μl was reverse transcribed with the ImProm-II Reverse Transcriptase cDNA Synthesis Kit (Promega) according to the manufacturer's instructions. The resulting cDNA reaction mix was then diluted 20 times in double-distilled water. Real-time quantitative PCR was performed with the SYBR Green mix (Roche) according to the manufacturer instructions.

The specific primers were as follows: Sema4B primers, forward: 5'-GTGGGACGTAACTCCTTCCA-3', reverse: 5'-AGGTTGCTCAAGTGGAATCG-3'; GAPDH primers, forward: 5'-TCAATGAAGGGGTCGTTGAT-3', reverse: 5'-CGTCCCGTAGACAAAATGGT-3'; ALDH1L1, forward: 5'-GACAAGGATGGGAAAGCAGA-3', reverse: 5'-CCACCGAGGGAACTTAAACA-3'; GFAP, forward: 5'-CAGCCTCAGGTTGGTTTCAT-3', reverse: 5'-GGAGAGGGACAACTTTGCAC-3'; vimentin primers, forward: 5'-GAAATTGCAGGAGGAGATGC-3', reverse: 5'-GTGCCAGAGAAGCATTGTCA-3'; and lipocalin-2 (LCN-2) primers, forward: 5'-CCTGGAGCTTGGAACAAATG-3', reverse: 5'-ATGTCACCTCCATCCTGGTC-3'.Amplicon purity and size were verified by melt curve analysis and gel electrophoresis. Each sample was normalized relative to GAPDH and ALDH1L1 with similar results.

#### Immunofluorescence analysis

Brains were fixed in 4% paraformaldehyde, followed by incubation in 30% sucrose for at least 24 h. Tissue sections (25 μm thick) were incubated for 1 h in a blocking solution consisting of 0.3% Triton X-100, and 5% goat serum. This solution was used for the dilution of both primary and secondary antibodies. Sections were incubated in the primary antibody overnight (16 h). Slides were then washed three times (5 min each) with PBS and visualized with Cy3- and Cy5-labeled secondary antibodies and coverslipped. Every fifth section was stained and analyzed with the different antibodies. At least seven sections were analyzed for each mouse. Each section was analyzed by confocal microscopy in 5 μm optical sections, and the noninjured side was used to determine background levels.

Sections were analyzed using a laser-scanning confocal microscope (FV1000; Olympus). The *z*-stack images were acquired with a 20× objective, 0.5 μm steps, and confocal acquisition software (FluoView, Olympus).

### Sema4B knockdown

For Sema4B knockdown experiments, we used MISSION shRNA lentiviral vectors. For mouse Sema4B, we used TRCN0000112290 (shRNA1) and TRCN0000112293 (shRNA2). Nontarget shRNA (sh-scramble) was used as a control.

### Lentivectors and lentiviral preparation

The cDNA encoding mSema4B was mutated to replace amino acids 666-673 (MDQK*NQ*RD) with the myc tag sequence (MEQKLISEE DL). This location has low conservation between mice and humans. It is located between the Ig domain and the transmembrane domain. This myc tag Sema4B was also mutated to generate cDNA encoding Sema4B in which Ser825 was replaced by alanine (S825A) or aspartic acid (S825D). All three alternative forms of Sema4B were cloned into a pLenti6.2 vector and cotransfected into HEK293 cells with expression vectors containing all the genetic elements required for pseudoviral particles. The titers of the different viruses used in rescue experiments were measured.

### Fusion proteins

The Fc fusion protein of the ectodomain of mouse Sema4B (amino acids 1-700) was cloned into PsxFc2 vector upstream of the hinge region of human IgG1-Fc. Fc protein was used as control. Fusion proteins were transiently expressed in 293 cells and were collected 72 h later. Concentrations of the Fc fusion proteins were evaluated by Western blot with anti-hIgG antibody.

### Astrocyte cell culture

Mouse primary cortical astrocytes were cultured essentially as described previously, with minor modifications (Schildge et al., 2013). Briefly, cerebral hemispheres were aseptically removed from newborn (2-d-old) pups, and the cortices were incubated in 0.25% trypsin for 10 min at 37°C. Tissues were then mechanically dissociated and resuspended in DMEM containing 15% fetal bovine serum (FBS) and antibiotics. Cells were grown in precoated poly-l-lysine 140 cm^2^ culture plates in DMEM with 10% FBS for 3 d. The medium was replaced on day 3 with modified DMEM (with d-valine instead of l-valine) and dialyzed serum. The new selection medium did not support the growth of fibroblasts and inhibited their proliferation. Astrocytes reached confluence between day 7 and day 10. Each culture preparation was tested for astrocyte purity by staining with anti-S100β. All cultures were between 98% and 100% positive for this marker.

### Adult astrocyte isolation

Cortices of a few adult mice were dissected and dissociated using papain and DNase, as described previously (Moussaud and Draheim, 2010). Astrocytes were then purified using anti-GLAST MicroBeads (Miltenyi Biotec) according to the manufacturer protocol, and protein was immediately extracted to be used in Western blot analysis.

### Flow cytometry of adult astrocytes

A biopsy sample of 3 × 3 mm around the site of injury in the cortex was removed 7 d following injury and PBS perfusion, and was dissociated using papain. The cells were stained with anti-GLAST and anti-BrdU, and analyzed by flow cytometry (MACSQuant Analyzers, Miltenyi Biotec).

### Statistical analysis

Values are presented as the mean ± SEM. A *p* value of <0.05 was considered to be significant. Statistical analysis was performed using a one-tailed or two-tailed Mann–Whitney test. Where relevant, *p* values were adjusted for multiple comparisons in accordance with the Bonferroni procedure; overall *p* values for the different injury experiments were then computed from these adjusted *p* values using Fisher’s χ^2^ test for combined probabilities. Asterisks indicate the following: *p < 0.05, **p < 0.001, and ***p < 0.0001.

## Results

### Sema4B is expressed in injured adult cortex astrocytes

**Figure 1:**
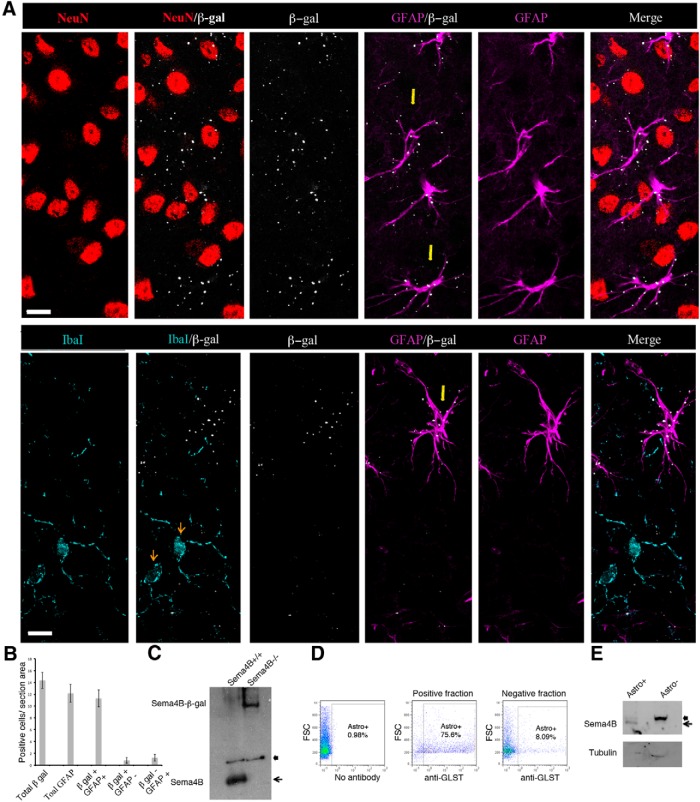
Sema4B is expressed by astrocytes *in vivo*. ***A***, Sema4B-expressing cells were identified in the mouse cortices using anti-β-gal antibody (which recognizes the Sema4B−β-gal fusion protein). In each set of experiments, Sema4B^+/−^ mice without injury, and 1, 3, and 7 d postinjury were analyzed. A *z*-stack of four optical sections is presented after staining with anti-NeuN, anti-GFAP, and anti-β-gal (top); or anti-Iba1, anti-GFAP, and anti-β-gal (bottom). The representative result 7 d after injury is shown. Yellow arrows mark examples of astrocytes, and orange arrows mark examples of microglia. Note the close proximity of the β-gal staining to the GFAP-positive staining but not to NeuN- or IbaI-positive staining. Scale bars, 10 μm. Multiple cells in a number of sections from three different mice were analyzed at each time point. ***B***, Quantitation represented as the number of positive cells per section for cells stained with anti-β-gal and anti-GFAP is shown. ***C***, Immunoblots of protein samples extracted from Sema4B^+/+^ and Sema4B^−/−^ mouse brains are presented. Arrow marks the Sema4B-specific signal, and arrowhead marks a nonspecific band recognized by this antibody. ***D***, Cortical tissue was dissociated and separated on MACS columns using ACSA-2-beads. Cell samples after the separation of cells were analyzed by FACS using anti-ACSA2-APC antibody. ***E***, Immunoblots of protein samples extracted from the positive (ACSA2^+^) and negative (ACSA2^−^) fractions. The blot was probed with anti-Sema4B antibody. Note that in the positive fraction there are two bands (the bottom one is the Sema4B signal), while in the negative fraction only the top band, which is a nonspecific band, appears (marked with an arrowhead).

We used a cortical stab wound lesion model to investigate the possible role of Sema4B in brain injury. We first decided to map the types of cells expressing Sema4B in the cortex using a secretory trap mouse (Friedel et al., 2005). The β-gal reporter gene in this mouse was fused to Sema4B (forming a Sema4B-β-gal chimeric protein, which is retained within an intracellular compartment). Immunofluorescence staining was used to analyze heterozygous Sema4B mice for β-gal reporter expression *in vivo*. We examined the cortex before, and 1, 3, and 7 d following stab wound injury, and at different distances from the site of injury, to include all cell types that may express Sema4B as a result of the injury. We costained for the β-gal reporter (which recognizes the Sema4B fusion protein), GFAP (an astrocyte marker), and NeuN (a neuronal marker), using specific antibodies. We also costained for the β-gal reporter, GFAP, and Iba1 (microglia marker). For each cell type and for each time point, we monitored hundreds of cells in different sections prepared from three different mice. Sema4B was never detected together with NeuN or Iba1 in any of the β-gal-positive cells (Fig. 1*A*). In contrast, most β-gal-positive cells (at days 3 and 7) were also GFAP positive, indicating that in the mouse cortex, Sema4B is mostly expressed by astrocytes. To quantify this observation, we counted the number of β-gal-positive and GFAP-positive cells 7 d after injury (Fig. 1*B*). Most GFAP-positive cells (∼90%) are also β-gal positive; GFAP-positive staining was undetectable in only 5.6% of the β-gal-positive cells. These cells may still be astrocytes, but with low or no detectable levels of GFAP expression.

We also looked for alternative methods to identify the types of cells expressing Sema4B. We tested a number of commercially available antibodies raised against Sema4B. None of the antibodies were useful in immunohistochemistry, but one of the antibodies worked well in Western blots. We validated this antibody using Sema4B^+/+^ and Sema4B^−/−^ brain lysates (Fig. 1*C*). Sema4B was detected in wild-type mice at its expected size (∼110 kDa; an additional nonspecific band just above this band was also detected). In Sema4B^−/−^ mice, Sema4B was detected at a size of ∼190 kDa, corresponding to the expected size of the Sema4B−β-gal fusion protein. We therefore used this antibody to confirm the types of cells expressing Sema4B. We first isolated astrocytes from mice cortices using an anti-EAAT1/GluT-1/GLAST (ACSA-2) magnetic cell separation kit. Using allophycocyanin (APC)-labeled ACSA-2 antibody in FACS analysis, we determined that the positive astrocyte fraction consisted of at least 75% astrocytes, while the negative fraction included only 8% astrocytes (Fig. 1*D*). This is likely to be an underestimate of the GLAST-bearing cells in the positive fraction, since the ACSA-2 beads may reduce the efficiency of ACSA-2-APC binding during the FACS analysis. We then evaluated both negative and positive cell populations by Western blotting. The proteins extracted from these cell populations were tested with antibodies against Sema4B and tubulin (Fig. 1*E*). Sema4B was detected in the astrocyte fraction, but not in the nonastrocyte fraction of cells, indicating that Sema4B expression in the cortex is restricted to the astrocyte cell population.

### Astrocyte activation is modified in Sema4B^−/−^ mutant mice

To investigate a possible role of Sema4B in astrogliosis, we analyzed cortical stab wound lesions of adult Sema4B^−/−^ mutants and their littermates (Sema4B^+/−^ and Sema4B^+/−^ or Sema4B^+/+^). We focused on day 7 postinjury, by which activation of astrogliosis is known to reach its peak (Robel et al., 2011).

**Figure 2:**
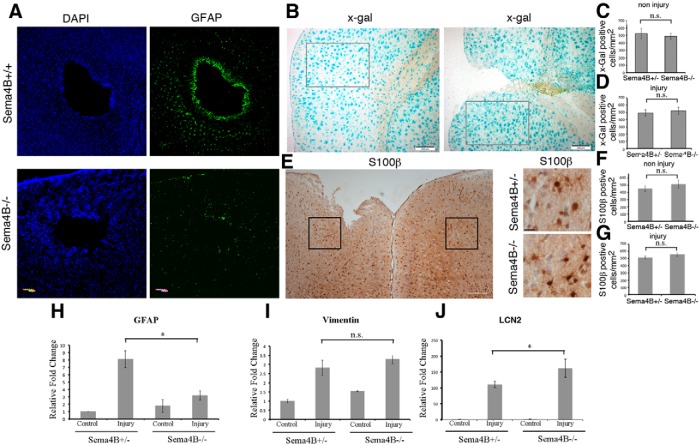
Astrocyte activation profile is modified in Sema4B^−/−^ mutant mice. ***A***, Representative examples of a horizontal section of wild-type (*n* = 7) and Sema4B mutant cortices were stained with GFAP (green) and Hoechst stain (blue) 7 d after injury. Note the reduced activation indicated by GFAP staining near the site of injury in the mutant mouse. Scale bar, 100 μm. ***B***, a representative coronal section of Sema4B^+/−^ with or without injury stained with X-gal histochemistry. Scale bar, 200 μm. The gray boxes mark the area of counting that was used in all sections. ***C***, Quantitation of X-gal-positive cells/area in the noninjured cortex of Sema4B^+/−^ and Sema4B^−/−^ mice. ***D***, Quantitation of the number of X-gal-positive cells per area in the injured cortex of Sema4B^+/−^ and Sema4B^−/−^ mice. ***E***, Representative example of cortex after injury in low magnification, stained with anti-S100β. The black boxes mark the area of counting (in ***F*** and ***G***). Scale bar, 200 μm. Representative example of Sema4B^+/−^ and Sema4B^−/−^ mice near the site of injury stained with S100β is also shown. Scale bar, 20 μm. Note that the typical astrocyte hypertrophy is detected in both Sema4B^+/−^ and Sema4B^−/−^. ***F***, Quantitation of the number of S100β-positive cells per area in the noninjured cortex of Sema4B^+/−^ and Sema4B^−/−^ mice. ***G***, Quantitation of the number of S100β-positive cells per area in the injured cortex of Sema4B^+/−^ and Sema4B^−/−^ mice. ***H−J***, Relative mRNA levels were measured using real-time PCR analysis (mean ± SEM) of cortical tissue at the site of injury 1 d postinjury (***I***: LCN2, Sema4B^+/−^, *n* = 4; Sema4B^−/−^, *n* = 5) and 7 d postinjury (***J***: GFAP; ***H***, vimentin; *n* = 5; Sema4B^+/−^, *n* = 5; Sema4B^−/−^, *n* = 6). *GFAP* LCN-2 and vimentin in each sample were normalized relative to GAPDH (similar results were obtained when samples were normalized to ALDH1L1). Note the change in postinjury astrocyte activation profile in the Sema4B^−/−^ cortex (low GFAP, normal activation of vimentin, and higher activation than normal for LCN2. For GFAP, *p* = 0.0081; for LCN2, *p* = 0.0357.

A hallmark of astrocyte activation is the upregulation of GFAP (Kindy et al., 1992; Yamashita et al., 1996). Expression of GFAP in astrocytes of the intact adult mouse cortex is very low in the absence of astrogliosis, but is significantly increased after CNS injury (Kindy et al., 1992; Yamashita et al., 1996). To monitor the activation of astrocytes, we first tested the expression of GFAP by immunofluorescence staining. Staining of horizontal cortical sections from unilaterally injured Sema4B mice revealed elevated GFAP levels almost exclusively on the injured side (both Sema4B^+/+^ and Sema4B^+/−^ were indistinguishable). Strong astrocyte staining was also observed around the stab wound site, with GFAP-positive processes extruding from astrocytes to form the astroglial barrier. GFAP staining of the Sema4B^−/−^ mutant mice, though present, was significantly reduced (Fig. 2*A*). This reduced GFAP activation can be the result either of limited GFAP activation or reduced astrocyte number. To address this point, we stained Sema4B^+/−^ and Sema4B^−/−^ mice cortices with or without injury with X-gal (Fig. 2*B*). We tested the cell density of X-gal (Sema4B-positive cells per square millimeter), comparing Sema4B^+/−^ and Sema4B^−/−^ mice cortices without injury. There was no significant difference in X-gal-positive cell density between genotypes, indicating that Sema4B-expressing astrocytes (in Sema4B^+/−^ mice) are not lost in Sema4B^−/−^ mice (Fig. 2*C*). We also tested the density of X-gal-positive cells in both Sema4B^+/−^ and Sema4B^−/−^ mice cortices after injury. Again, no significant difference between the two genotypes after injury was detected (Fig. 2*D*). We then stained for S100β, another astrocyte marker, and tested the density of cells with or without injury (Fig. 2*E*). Again, no significant change in the number of S100β cells was detected (Fig. 2*F*,*G*), demonstrating that the lack of GFAP activation is not the result of a reduced number of astrocytes (we did not try to colocalize the X-gal and the S100β since the conditions of the two methods are incompatible).

Real-time PCR was used to assay *GFAP* expression in mRNA extracted from cortical tissue around the stab wound to obtain more quantitative information on astrocyte activation levels. *GFAP* expression in the noninjured brains of Sema4B^+/−^ and Sema4B^−/−^ mutant mice was similar. In contrast, the expression of *GFAP* mRNA in Sema4B^−/−^ 7 d after stab wound injury was ∼2.5-fold lower than in the Sema4B^+/−^ mice (Fig. 2*B*). To test whether GFAP is the only gene affected in Sema4B^−/−^ mice, we also tested the expression of two additional markers of astrocyte activation (vimentin and LCN-2). Vimentin, another intermediate filament protein, which is induced by some reactive astrocytes (Galou et al., 1996), was increased to the same extent in the injured cortices of the Sema4B^+/−^ and Sema4B^−/−^ mutant mice (Fig. 2*I*). In contrast, LCN2, an iron-trafficking protein shown to be specifically induced in astrocytes following inflammation and stroke (Zamanian et al., 2012), was also induced following stab wound injury, but activation in Sema4B^−/−^ mice was ∼1.5 times higher compared to that in Sema4B^+/−^ mice (Fig. 2*J*). These results suggest that astrocytes are activated following cortical injury in Sema4B^−/−^ mice, although their activation profile differs from that of wild-type astrocytes.

### Astrocyte proliferation is reduced in Sema4B mutant astrocytes *in vivo*

**Figure 3:**
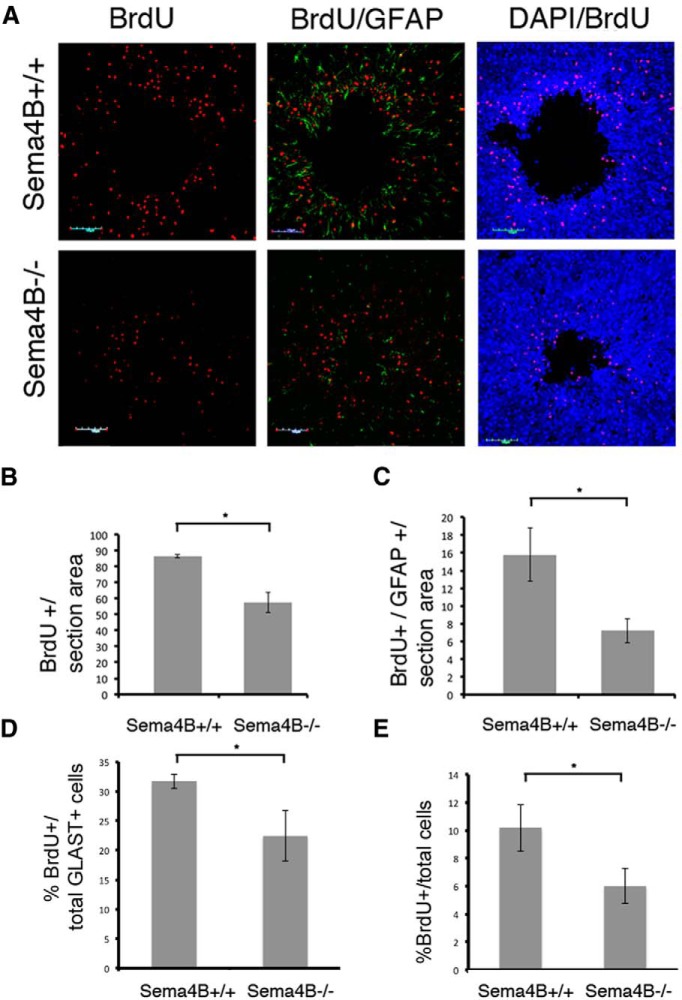
Astrocyte proliferation *in vivo* is reduced in Sema4B^−/−^ mutant mice. ***A***, Representative cortical slices in Sema4B^+/+^ and Sema4B^−/−^ mutant mice 7 d postinjury. The panel shows proliferating cells labeled with BrdU (red), activated astrocytes stained with anti-GFAP (green), and total cells labeled with DAPI (blue). Scale bars, 100 μm. ***B***, ***C***, Quantification of the total number of proliferating cells (BrdU^+^; ***B***) and of proliferating astrocytes (GFAP^+^/BrdU^+^; ***C***). ***D***, Cell proliferation evaluation by flow cytometry analysis of the cortical injury site. The cortical tissue site was dissociated, and analyzed using anti-BrdU and anti-GLAST antibodies. Results in ***B−E*** are given as the mean ± SEM. ***B***, *p* = 0.032; ***C***, *p* = 0.041; ***D***, *p* = 0.0285; and ***E***, *p* = 0.0385.

A restricted number of astrocytes proliferate in response to cortical injury (Bardehle et al., 2013). To monitor the effect of Sema4B on injury-induced proliferation, we injected Sema4B^−/−^ mutant mice and wild-type littermates with BrdU, a marker of cell proliferation, on postinjury days 3, 5, and 7, a strategy that ensures that most of the proliferating cells will be labeled (cell counts on day 7 are shown in Fig. 3). There were significantly fewer BrdU-positive cells per section area near the site of injury (indicating significantly less cell proliferation) in the Sema4B null mice than in the wild-type mice (Fig. 3*B*). There was an even greater percentage reduction in the BrdU-positive cells that were also positive for GFAP in the mutant mice than in the wild-type mice (Fig. 3*C*), suggesting that most of the cells that exhibited a decreased rate of proliferation in the absence of Sema4B were astrocytes.

We cannot, however, rule out that the observed difference between the total cell count and the GFAP-positive cell count is not simply because GFAP immunostaining is reduced in the Sema4B mutant mice. To test the proliferation of astrocytes in a manner independent of GFAP, and to overcome the limitation of immunohistochemistry, we also tested proliferation using flow cytometry analysis. In these experiments, the astrocytes were labeled with anti-GLAST and anti-BrdU (again injected on days 3, 5, and 7). Consistent with the immunostaining experiments, we detected a reduction in total cell proliferation, and specifically in astrocyte proliferation. Although reduction in astrocyte proliferation significantly contributes to the reduction in cell proliferation, it is possible that other, as yet unidentified, cell types are less proliferative after injury in the cortices of Sema4B^−/−^ mice.

### Proliferation of Sema4B^−/−^ astrocytes *in vitro* is reduced

**Figure 4:**
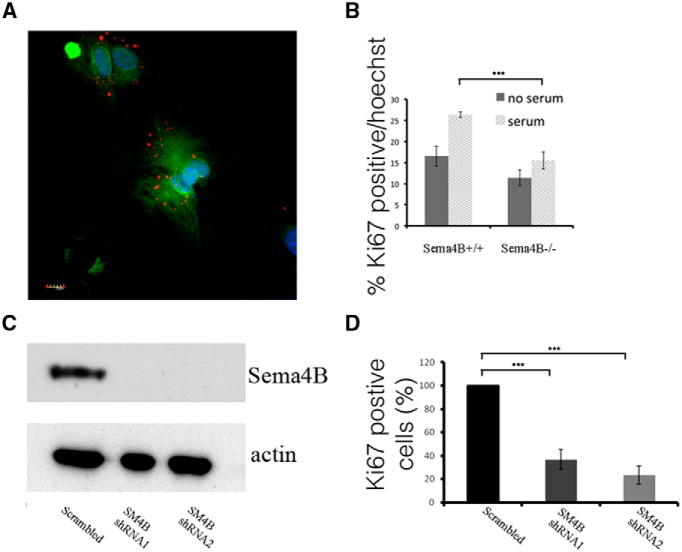
Both short-term and long-term inhibition of Sema4B reduces the proliferation of astrocytes *in vitro.* ***A***, Sema4B-expressing astrocytes in culture were identified using anti-β-gal antibody and GFAP. Scale bar, 10 μm. ***B***, Quantification of proliferating cultured astrocytes (percentage of Ki-67-positive cells/Hoechst stain) is shown. For this experiment, astrocytes from wild-type and mutant mice were serum starved for 3 d before being stimulated with serum and tested for proliferation. ***C***, ***D***, Short-term inhibition of Sema4B in primary astrocytes from wild-type mice is shown. ***C***, Representative Western blot showing reduction in the levels of Sema4B, 72 h after infection with two different anti-Sema4B shRNAs or a scrambled shRNA (*n* = 3). ***D***, Quantification of proliferating shRNA-treated wild-type astrocytes (percentage of Ki-67-positive cells/Hoechst stain) is shown. Results in ***B*** and ***D*** are the mean ± SEM of four independent experiments in each case. ***B***, *p* = 2.5^E-9^; and ***D***, *p* = 0.000153 and *p* = 0.000704.

To further investigate the role of Sema4B in astrocytes under more controlled conditions, we measured astrocyte proliferation *in vitro*. As a first step, we tested whether Sema4B is expressed by astrocytes in culture. As shown in the cortex, astrocytes obtained from Sema4B^+/−^ mice are also positive for β-gal (Fig. 4*A*). After confirming that Sema4B is expressed by astrocytes in culture, we compared the proliferation of Sema4B^+/−^ and null astrocytes *in vitro*. Astrocytes were serum starved for 3 d, and then stimulated with serum for 16 h. Staining of the cells with Ki-67 antigen, a marker of cell proliferation, clearly showed that astrocytes from Sema4B-deficient mice are less responsive to serum-induced proliferation than those from the heterozygous control (Fig. 4*B*).

To exclude the possibility that the effect on astrocyte activation in the Sema4B mutant astrocytes is caused either by gain-of-function by the fusion protein (Sema4B−β-gal) or because a pre-established developmental defect underlies the abnormal response of Sema4B^−/−^ astrocytes, we inhibited Sema4B expression in the short term by infecting wild-type primary astrocytes with two different shRNAs targeting Sema4B. Both shRNAs reduced the protein levels of Sema4B to almost undetectable levels compared to scrambled shRNA (Fig. 4*C*). As in the case with astrocytes from Sema4B^−/−^ mutant mice, shRNA knockdown of Sema4B expression also reduced cell proliferation (Fig. 4*D*). To validate the shRNA experiment, we performed a rescue experiment in which we infected astrocytes with both Sema4B expression vector and shRNA targeting Sema4B. Unfortunately, validation of our shRNA experiments was not possible since astrocytes were not viable after selection for both viruses, probably because the infection efficiency was not high enough. Nevertheless, since both the shRNA and the Sema4B mutant astrocytes show very similar results, it is most likely that Sema4B is indeed required for astrocyte proliferation.

### Sema4B expression is not regulated at the level of mRNA or protein expression

**Figure 5:**
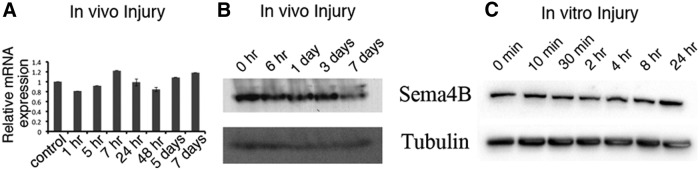
Sema4B is not regulated at the level of mRNA or protein. ***A***, ***B***, Sema4B RNA (***A***) and protein (***B***) expression in a biopsy sample obtained from cortex near the site of injury. ***A***, Real-time PCR assay of Sema4B mRNA in the cortex at different time points after injury (*n* = 3). ***B***, Representative Western blot of protein samples at different time points after injury are shown (*n* = 4). ***C***, Protein was extracted from confluent astrocyte cultures at different time points after scratch wound injury and analyzed with anti-Sema4B. The same blots were also tested for tubulin.

Our results thus far indicate that Sema4B is needed for astrocyte activation and proliferation following injury. It is therefore possible that Sema4B expression would be increased following injury. We first tested whether Sema4B is regulated at the level of mRNA expression by measuring the levels of Sema4B mRNA at different time points after cortical injury (Fig. 5*A*). The results indicated that Sema4B is not regulated at the level of mRNA, prompting us to study whether Sema4B may be regulated at the protein level. To test this possibility, we extracted proteins from cortical biopsy samples from the site of injury at different time points. The protein levels of Sema4B were also not significantly changed (Fig. 5*B*). Finally, we did not detect any changes in the protein levels of Sema4B in astrocytes in response to *in vitro* injury (Fig. 5*C*). Although globally Sema4B does not appear to be regulated by injury, it is still possible that Sema4B is upregulated in some cells in the cortex, while in other cells it is downregulated. However, histochemical staining with X-gal (Fig. 2*B*) does not support this possibility. Thus, Sema4B is regulated neither at the RNA nor at the protein level following injury.

### Sema4B is phosphorylated at S825 following injury or growth factor stimulation

**Figure 6:**
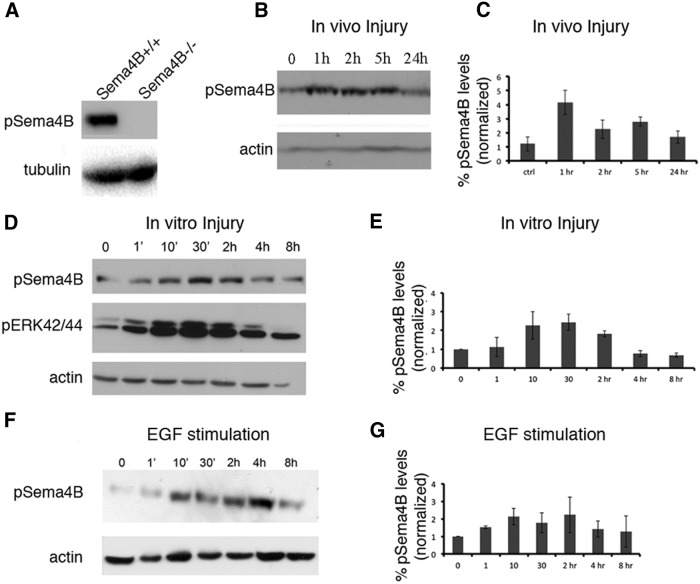
Sema4B is phosphorylated at Ser825 after growth factor stimulation or injury. ***A***, Immunoblots of protein samples extracted from Sema4B^+/+^ and Sema4B^−/−^ brains and tested with anti-phospho-Sema4B and anti-tubulin are shown. ***B***, Protein extracted from biopsy samples from the site of the mouse cortical stab wound injury at different times after injury. Representative results are shown. ***C***, Densitometric analysis of the phospho-Sema4B/actin ratio at different times after cortical injury is shown (*n* = 4). ***D***, Western blots of protein samples were extracted from mouse cortical astrocytes at different times after mechanical injury. Representative Western blots obtained with anti-phospho-Sema4B at various time points are shown. The same blots were also tested for p-ERK1/2 and actin. ***E***, Quantification of the phospho-Sema4B/actin ratios obtained in the mechanical injury experiments are shown (*n* = 4). ***F***, Western blots of protein samples were extracted from mouse cortical astrocytes at different times after stimulation with 100 ng/ml EGF. ***G***, Densitometric analysis of the phospho-Sema4B/actin ratio at different times after stimulation with 100 ng/ml EGF (mean ± SEM of three independent experiments is shown for each time point). Results in ***B***, ***D***, and ***E*** are given as the mean ± SEM of four independent experiments.

Although we have not detected injury-related regulation of Sema4B expression, our genetic evidence indicates that it has an important role in astrocyte activation after injury. We therefore considered the possibility that Sema4B is regulated post-translationally. An earlier study (Moritz et al., 2010) showed that Sema4B is phosphorylated at serine 825 in some cancer cell lines stimulated with growth factors such as EGF. To examine whether Sema4B is phosphorylated after injury, we assayed Sema4B phosphorylation at different times after cortical stab wound injury in Sem4B^+/+^ mice using a phosphorylation-specific antibody directed against serine 825 of Sema4B. First, the specificity of the antibody was tested using brain tissue from Sema4B^+/+^ and Sema4B^−/−^ mice. Since β-gal and a stop codon are inserted upstream from the transmembrane domain, we expected that phospho-Sema4B would not be present in mutant mice. Indeed, we found that the antibody is highly specific and identifies phospho-Sema4B only in wild-type mice (Fig. 6*A*). Using this antibody, we detected rapid phosphorylation of Sema4B, with a peak 1-5 h following stab wound injury (Fig. 6*B*,*C*).

Astrocytes isolated *in vitro* were used to study this phosphorylation more carefully. A rapid increase in Sema4B phosphorylation was also detected in confluent primary astrocytes following a scratch wound (Fig. 6*D*,*E*). As a positive control, we monitored ERK1/2 phosphorylation, which has been shown to be activated in scratch wound experiments (Mandell et al., 2001). The kinetics of ERK1/2 and Sema4B phosphorylation was very similar. It has been shown in the past that the EGF receptor (EGFR) is activated in astrocytes following injury and can affect astrocyte response (Yang et al., 2011). We therefore tested the effects of EGF on Sema4B phosphorylation. As in the model of injury, primary astrocytes stimulated with EGF were rapidly phosphorylated at this site (Fig. 6*F*,*G*). These results indicate that the regulation of Sema4B may occur through phosphorylation at serine 825.

### Sema4B does not function as a ligand in astrocyte proliferation

**Figure 7:**
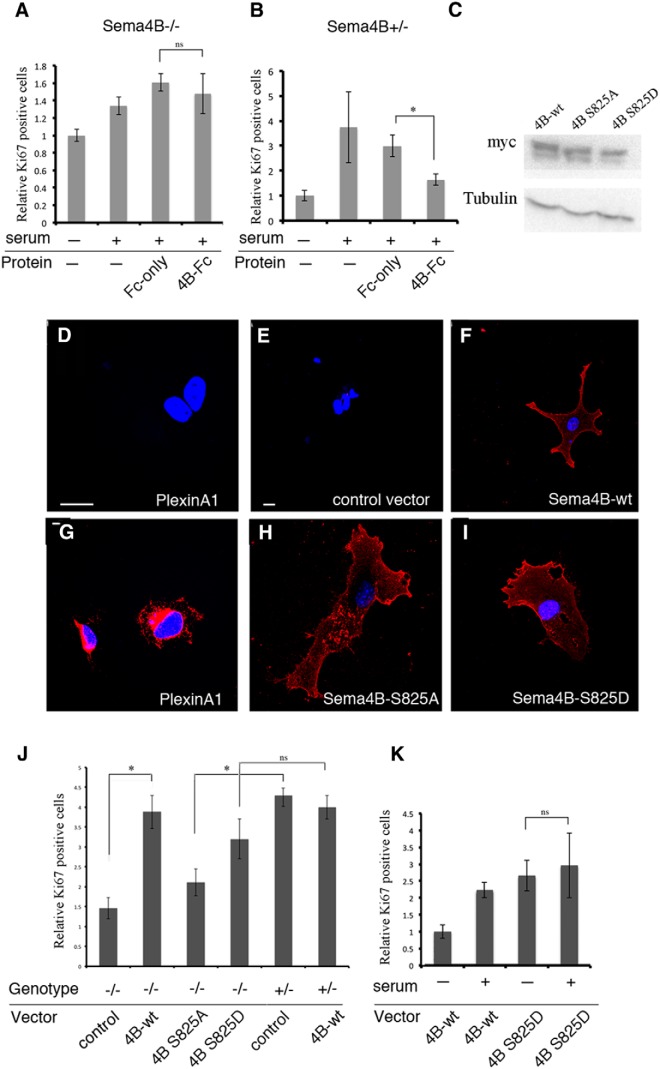
Sema4B phosphorylation at S825 is critical for its function. ***A***, ***B***, Astrocytes from Sema4B^−/−^ (***A***) or Sema4B^+/−^ (***B***) mice were incubated with serum-deficient medium for 3 d. Astrocytes were then incubated for an additional 16 h with 10% serum and recombinant proteins (50 ng/ml Fc-only or Sema4B-Fc). Note that Sema4B-Fc was not able to rescue the serum-induced component of Sema4B^−/−^ mouse astrocytes. ***C***, ***E***, ***F***, ***H***, ***I***, Astrocytes isolated from Sema4B^+/−^ or SemaB^−/−^ mutant wild-type mice were infected with lentiviral vectors expressing empty vector, wild-type (full-length) Sema4B, or point mutations that abolish Sema4B phosphorylation. Astrocytes were selected with Blasticidin until all noninfected astrocytes died. ***C***, Western blots of protein samples extracted from the infected astrocytes and tested for myc tag expression are shown. ***D***, ***G***, HEK293 cells transfected with PlexinA1 tagged with myc at the intracellular domain with (***G***) or without (***D***) detergent are shown. Scale bars, 20 μm. ***E***, ***F***, ***H***, ***I***, Representative astrocytes infected with the different vectors and stained with an anti-myc tag in the absence of detergent are also shown. Scale bars, 10 μm. ***J***, ***K***, Sema4B^−/−^ (***J***) and Sema4B^+/−^ (***J***, ***K***) astrocytes infected with the different Sema4B mutants were incubated in serum-deficient medium for 3 d before being stimulated for 16 h with 10% serum. Their proliferation was monitored by expressing Ki-67-positive astrocytes as a fraction of the total number of astrocytes in the same fields. Results are given as the mean ± SEM of four independent experiments. ***B***, *p* = 0.0247; ***J***, *p* = 0.0247.

Although Sema4B phosphorylation is more consistent with a receptor or signaling molecule, semaphorins in general, and Sema4B in particular, are known to function as ligands. The best example for this is the activity of Sema4B in the immune system. In this system, the Sema4B ectodomain has been shown to be necessary and sufficient to rescue the Sema4B^−/−^ phenotype in the immune system (Nakagawa et al., 2011). To test whether Sema4B functions as a ligand in our system, we examined whether the ectodomains of Sema4B (Sema4B-Fc) can rescue the proliferation response of Sema4B^−/−^ astrocytes to serum stimulation (Fig. 7*A*). Sema4B-Fc was not able to rescue the proliferation defect of astrocytes, implying that Sema4B may function as a receptor. If this were the case, we speculated that the ectodomains of Sema4B might act as a competitive inhibitor on wild-type astrocytes. To test this possibility, we repeated the same experiment, this time using wild-type astrocytes. Consistent with this idea, Sema4B-Fc reduced the serum-induced proliferation response (Fig. 7*B*). Based on these experiments, we considered the possibility that the intracellular domain of Sema4B is critical for its activity.

**Table 1:** *Post hoc* power calculations for *m* independent experiments with Bonferroni correction *k*

Figure	Panel	Data structure	Type of test	Power
2	*C*	Normality not assumed	Mann-Whitney	55.9
	*D*	Normality not assumed	Mann-Whitney	53.2
	*F*	Normality not assumed	Mann-Whitney	61.7
	*G*	Normality not assumed	Mann-Whitney	10.7
	*H*	Normality not assumed	Mann-Whitney	12.3
	*I*	Normality not assumed	Mann-Whitney	2.6
	*J*	Normality not assumed	Mann-Whitney	12.6
3	*B*	Normality not assumed	Mann-Whitney	21.5
	*C*	Normality not assumed	Mann-Whitney	63.3
	*D*	Normality not assumed	Mann-Whitney	36.4
4	*B*	Normality not assumed	Mann-Whitney	100
	*D*	Normality not assumed	Mann-Whitney	100100
7	*A*	Normality not assumed	Mann-Whitney	37.1
	*B*	Normality not assumed	Mann-Whitney	90
	*J*	Normality not assumed	Mann-Whitney	100100
	*K*	Normality not assumed	Mann-Whitney	99.9100

From Fisher's χ^2^ test for combined probabilities, we have that χ^2^ (df = 2*m*) ∼ –2ln(*p*1p2…*pm*), where *pi* is the *p* value for the *i*th independent experiment. The *post hoc* expected value of χ^2^ is just the df + the noncentrality parameter (λ). Thus, for three independent experiments, say λ is given by –2ln(p1p2p3) – 6. The power can then be obtained directly using G*Power 3 software with α = 0.05/k.

### Sema4B phosphorylation at S825 is required for astrocyte proliferation

Since Sema4B undergoes rapid phosphorylation after injury or growth factor stimulation at the intracellular domain, we examined whether such phosphorylation is important for the activity of Sema4B. We generated lentiviral vectors expressing either wild-type (full-length) Sema4B or a phosphorylation-resistant Sema4B, in which serine 825 is mutated to alanine, Sema4B^S825A^, and Sema4B^S825D^, a mutant form of Sema4B that probably mimics constitutive phosphorylation. We used the lentiviral vectors expressing either Sema4B mutant or wild-type proteins to infect astrocytes from Sema4B^+/−^ and Sema4B^−/−^ mice, and monitored cell proliferation by Ki-67 staining. To make sure that the mutations do not affect the expression or localization of Sema4B, we added a myc tag to the extracellular domain of Sema4B (for details, see Materials and Methods) to monitor the level of protein expression as well as cellular localization. All three Sema4B vectors resulted in similar expression, as shown by Western blots (Fig. 7*C*). To test whether Sema4B overexpression mutants are present correctly on the membrane, we used the localization of the myc tag in the extracellular domain to monitor expression in the cells without detergent, assuming that the myc tag would not be detected inside the cells. To check this assumption, we transfected HEK293 cells with PlexinA1, which contains a myc tag at the intracellular domain of the protein. The cells were stained with (Fig. 7*G*) or without (Fig. 7*D*) detergent. The results show that in the absence of detergent, the cells are negative for the myc tag. We then infected astrocytes with the different mutants and the empty vector, and stained the cells without detergent (Fig. 7*E*,*F*,*H*,*I*). The results clearly show that all Sema4B mutants are expressed correctly on the membranes of the astrocytes.

We subsequently used these vectors to test whether Sema4B function can be rescued in Sema4B mutant astrocytes. As shown in Figure 7*J*, the Sema4B^S825A^ mutant was not able to overcome the proliferation defect in Sema4B^−/−^ mutant astrocytes. In contrast, cell proliferation was restored in Sema4B^−/−^ mutant astrocytes expressing either the wild-type Sema4B or Sema4B^S825D^.

Thus far, we have found that Sema4B is phosphorylated following injury and that it needs to be phosphorylated in order to be able to function. However, can the phosphorylated form of Sema4B bypass the need for serum stimulation in order for astrocytes to proliferate? To answer this question, the lentiviral vectors were used to infect wild-type astrocytes with either wild-type Sema4B or constitutive phosphorylation mutant Sema4B^S825D^ (Fig. 7*K*). As expected, astrocytes infected with Sema4B wild-type vector showed low proliferation levels in the absence of serum and proliferated readily when serum was added. In contrast, astrocytes infected with Sema4B^S825D^ were highly proliferative (at least based on their Ki67 levels) independent of serum addition. This result suggests that phosphorylation of Sema4B at serine 825 can bypass the need for serum-dependent astrocyte proliferation.

## Discussion

Sema4B is a transmembrane type 4 semaphorin, which has been suggested to be expressed in astrocytes (Liu et al., 2006; Lovatt et al., 2007; Cahoy et al., 2008; Maier et al., 2011); however, the role of the *Sema4B* gene in astrocytes following brain injury is not known. Here, we show not only that astrocytes are likely the only cells in the adult cortex that express Sema4B, but also that Sema4B is essential for postinjury astrogliosis.

Cortical injury is known to induce expression of GFAP, a classic marker of astrocyte identity and activation, as well as astrocyte cell proliferation. Postinjury GFAP activation and astrocyte cell proliferation were significantly decreased in Sema4B^−/−^ mutant mice compared to wild-type mice. This is likely not indicative of reduced astrocyte numbers since the density of S100β-positive cells as well as X-gal (Sema4B)-positive cells did not differ significantly. This also does not appear to reflect the blocking of astrogliosis, since LCN2 and vimentin, other known markers of reactive astrocytes (Zamanian et al., 2012), are induced in both Sema4B^−/−^ and control mice. These results suggest that Sema4B is part of a specific signaling pathway required for astrocyte activation upon injury.

In this study, we used a global Sema4B-β-gal gene trap. In wild-type cells, Sema4B is located at the cell membrane. In contrast, in the Sema4B-β-gal gene trap, the chimeric protein formed is retained within an intracellular compartment. Although the expression of a chimeric protein in a new cellular localization may result in a gain of function, our results do not support this possibility. First, we did not see any difference between wild-type and heterozygous mice in any of the assays we tested, both *in vitro* and *in vivo*. Moreover, astrocyte proliferation is reduced in knockdown experiments, similar to the situation of astrocytes isolated from Sema4B mutant mice. Unfortunately, we were not able to verify the result of the knockdown with a rescue experiment, most likely due to technical difficulty. Nevertheless, although it is difficult to completely rule out the possibility that the shRNA result is somehow compromised, since a similar phenotype is observed in both mutant mice and shRNA Sema4B-treated astrocytes, this possibility is unlikely. Thus, the fusion protein is not likely to have a biological function.

An additional concern related to the use of this model is whether the effects on astrocyte activation we detected are a direct result of the mutation of Sema4B in astrocytes or in other cells. Although it is difficult to completely rule out the latter possibility, we do not think it is likely. First, we showed that in the cortex, Sema4B is most probably restricted to astrocytes, and, therefore, it is not likely that other cells are involved. In the context of the immune system, some T and B cells express Sema4B in the periphery (Nakagawa et al., 2011). Treatment with ectodomains of Sema4B represses secretion of IL-4 and IL-6 by immune cells, and activation of basophils in Sema4B^−/−^ mice is increased (Nakagawa et al., 2011). Thus, it is theoretically possible that the function of Sema4B in the immune system indirectly influences the astrocyte response. It is not known whether T or B cells, which could enter the cortex after injury, also express Sema4B. There is a remote possibility that a small number of T or B cells expressing Sema4B enter the cortex. However, even if we missed a small number of Sema4B-expressing immune cells, it is not likely to be the reason for the reduced activation of GFAP or the proliferation of astrocytes, since in Sema4B^−/−^ mice IL-6 and IL-4 secretion, and basophil activation are likely to be stronger. We would therefore expect to see increased inflammation as well as increased GFAP expression. Instead, we found that GFAP expression is reduced. Moreover, the number and profile of the immune cells in the cortex are not changed (data not shown). Finally, astrocytes *in vitro* show a similar phenotype to that of astrocytes *in vivo*. Thus, in the context of stab wound injury, it is almost certain that Sema4B expressed by astrocytes affects them directly.

### Regulation of Sema4B during injury

Sema4B does not appear to be regulated at the level of RNA or protein following injury. Instead, we found that Sema4B was phosphorylated at Ser825 following injury *in vitro* and *in vivo*. Recent work identified Ser825 as a site of Sema4B phosphorylation following mitogen stimulation in cancer cells (Moritz et al., 2010). This phosphorylation site is embedded within a sequence motif containing arginine residues at the 5' and 3' positions, typical of AGC kinases regulated by mitogenic stimuli such as Akt, RSK, and p70 S6K. Using Sema4B-null astrocytes in reconstitution experiments with Sema4B phosphorylation mutants, we found that Sema4B phosphorylation is critical for serum-induced astrocyte proliferation. Moreover, since constitutive phosphorylation mimicking of Sema4B reduces the need for serum-induced proliferation, it seems that phosphorylation of Sema4B is necessary and sufficient for this type of proliferative signal in astrocytes.

Interestingly, the regulation of Sema4B phosphorylation shortly after injury suggests that this protein is part of an early response of the astrocytes. Indeed, we see an indication of this in the overactivation of LCN2 in mutant mice just 24 h after injury. Nevertheless, most prominent markers of astrocyte activation, namely GFAP activation and proliferation, are induced days later. It is therefore tempting to speculate that Sema4B activity is needed to initiate a signaling cascade, which triggers a modified astrocyte activation profile. A similar profile is also observed with critical regulators of astrocyte activation such as Stat-3, which is phosphorylated shortly after injury (peak between 3 and 6 h after injury; Oliva et al., 2012), while the most dramatic effects on astrocyte activation are most prominent 7-14 d after injury (Okada et al., 2006).

### How does Sema4B regulate astrocyte activation?

Sema4B is a member of the semaphorin family, which comprises 20 evolutionarily conserved proteins classified as secreted (type 3) or transmembrane (types 46; Pasterkamp, 2012). Semaphorins commonly act as ligands that bind directly to plexins or neuropilins. For example, Sema4B negatively regulates basophil-mediated immune responses (Nakagawa et al., 2011). The fact that the ectodomain of Sema4B is sufficient for this effect on basophils provides evidence that Sema4B functions in this system as a ligand. However, at least type 6 semaphorins may also operate in a reverse-signaling mechanism as receptors, as shown for Sema6D/PlexinA1 (Toyofuku et al., 2004). Thus, it is possible that transmembrane semaphorins such as Sema4B may also function as receptors.

Does Sema4B function as a receptor or a ligand in modulating astrocyte activation? The dependence of astrocyte activation on Sema4B, shown in this study, as well as the fact that Sema4B is expressed in astrocytes, is consistent with the possibility that Sema4B functions in astrocytes as a signaling molecule, a receptor, or both. There may, however, be an indirect function in which Sema4B activates a receptor on adjacent cells (other astrocytes or other cell types) after injury, which in turn activates another ligand that can induce astrocyte proliferation. However, the fact that the ectodomains cannot rescue proliferation of mutant astrocytes and have a competitive inhibitor activity on wild-type astrocytes argues strongly for a receptor type of action. Moreover, the dependence of Sema4B on the phosphorylation of a key residue in its intracellular domain for its activity also supports the notion that Sema4B functions as a receptor or a coreceptor in this system. The receptor/ligand for Sema4B has not yet been identified. Since many members of these type 4 semaphorins act via PlexinB1 or B2, it has been suggested that Sema4B might activate one of these receptors (Pasterkamp, 2012). However, because Sema4B phosphorylation can occur downstream to receptor tyrosine kinase signaling, instigated by EGFR, platelet-derived growth factor receptor, or Met (Moritz et al., 2010), it is also possible that in this case Sema4B functions as a signaling molecule in which both binding of the ligand at the extracellular domain and RTK signaling are needed for the activation of signaling downstream of Sema4B.

### How does Sema4B function as a signaling molecule?

Our results suggest that the intracellular domain of Sema4B plays a critical role in astrocytes. This intracellular domain contains ∼100 aa. Except for the C-terminal regions, most of this sequence is not conserved within the semaphorin family (although it is quite conserved between species). The C-terminal region of Sema4B, as well as of Sema4C, Sema4F, and Sema4G, has a PDZ binding motif (Burkhardt et al., 2005). Sema4B activity in neurons has been suggested to involve PDZ-containing proteins via its PDZ-binding motif (Burkhardt et al., 2005). It is thus possible that one mechanism by which Sema4B interacts with other signaling molecules in astrocytes is through its PDZ binding motif.

The interacting proteomes in astrocytes are not yet known. Based on a large-scale PDZ binding selectivity screen across the mouse proteome, it seems that the PDZ binding domain of Sema4B has the potential to interact with a small number of PDZ domain proteins, among them the serine proteases HtrA1 and HtrA3, and the protein phosphatase PTPN13 (Stiffler et al., 2007). Interestingly, Sema4B phosphorylation occurs at serine 825, eight residues from the C-terminal sequence of Sema4B. Phosphorylation of amino acids at this position has been suggested to modulate PDZ binding functions (Boisguerin et al., 2007). Our data strongly suggest that Sema4B signals to significantly modulate the biology of astrocytes after CNS injury. The detailed regulatory mechanisms of this novel pathway and the impact on the recovery of the CNS after injury remain to be examined.

## Synthesis

The decision was a result of the Reviewing Editor Michael Fainzilber and the peer reviewers coming together and discussing their recommendations until a consensus was reached. A fact-based synthesis statement explaining their decision and outlining what is needed to prepare a revision is listed below. The following reviewers agreed to reveal their identity: Wilma Friedman and Avraham Yaron

Reviewer comments:

This study represents a potentially novel function for sema 4B in regulating astrocyte activation, however there are a number of concerns with the manuscript that need to be addressed:

1) Figure 1 examines the cell types in the cortex that express the sema 4B-ß-gal fusion protein and conclude that it is expressed exclusively in GFAP-positive astrocytes, however this is difficult to see in the images presented in figure 1a.

2) In figure 3 they examined astrocyte proliferation after the stab injury and show fewer BrdU-positive cells in the mice lacking sema 4B. As in figure 1, it is very difficult to ascertain the co-localization of BrdU with GFAP labeling at this magnification. Moreover, in figure 3c they indicate that there is less colocalization of BrdU with GFAP in the sema4B-/- mice, suggesting that there is a selective decrease in astrocyte proliferation. However, as they point out, since the sema 4B-/- mice showed a specific decrease in GFAP expression, it is surprising that they didn't use a different astrocyte marker, such as S100ß, whose expression was unaffected by the absence of sema4B, for this analysis.

3) What is the in vitro injury indicated in figure 5c?

4) The result with 4B S825D in figure 7j is unclear. Although the text indicates that both 4B-wt and S825D restored proliferation, the significance bar seems to indicate a difference between -/- with S825A and +/- control, not -/- with S825D. Additionally, they conclude from their experiment in figure 7k that Sema 4B phosphorylation at serine 825 is required for serum-dependent astrocyte proliferation, but the experiment only shows that this phosphorylation can bypass the need for serum, not that it's necessarily required.

5) It is not clear from their method for growing astrocyte cultures how pure their preparation of astrocytes is. Although they state that 98-100% of their cells are S100ß-positive, they have not stained for the presence of contaminating cells, such as microglia and oligodendrocyte progenitors, which are likely to be present since their method is only to dissociate and plate the cells in serum-containing media with no shaking to remove other cell populations as is usually required to obtain a pure astrocyte culture.

6) A general concern is that the phenotypes arise from developmental defects. The authors try to overcome this issue through the use of shRNAs and acute KD in vitro, but the results are puzzling and poorly controlled. There is a reduction of about 50-60% in the mRNA by the shRNAs while the heterozygous mice have no phenotypes. Importantly, protein reduction is not evaluated and the specificity of the shRNAs is not tested. Western blots of Sema4B are required in the KD experiments.

7) Following on from point 6 above, a rescue experiment of the shRNAs with an shRNA-resistant form of Sema4B is needed to verufy specificity of the shRNA results.

8) An optional addition to the data would be gene expression analysis of GFAP, Vimentin and LNC2, which were tested in vivo, in the non-phospho Sema4B infected astrocytes. This will indicate if the signaling from that site is contributing only to the proliferative response or also for astrogliosis associated gene expression.

## Author Response

Dear Dr. Fainzilber,

Thank you for the time you spent discussing our manuscript (eN-NWR-0078-14), and for the opportunity to resubmit it. We appreciate the constructive comments of the reviewers, as well as their specific questions and the issues they raised. We have modified the manuscript to address these issues. Below please find our responses to their specific points.

1) “Figure 1 examines the cell types in the cortex that express the sema 4B-ß-gal fusion protein and conclude that it is expressed exclusively in GFAP-positive astrocytes, however this is difficult to see in the images presented in figure 1a”.

To address this comment we replaced the photographs in Figure 1a and changed the color composite to make this figure clearer.

We would also like to add a few comments:

1) We do not claim that Sema4B is exclusively expressed in astrocytes because at least from the GFAP staining results, there is a small minority of cells that are positive for β-gal but not for GFAP (Fig. 1b). It is still possible that these cells are astrocytes that express very low levels of GFAP, but we did not rule out the possibility that another population of cells also expresses Sema4B.
2) MACS magnetic bead separation of wild-type astrocytes based on their GLAST expression (Fig. 1e) and monitoring of Sema4B protein itself independently support our conclusion that Sema4B is mostly expressed by astrocytes.

2) “In figure 3 they examined astrocyte proliferation after the stab injury and show fewer BrdU-positive cells in the mice lacking sema 4B. As in figure 1, it is very difficult to ascertain the co-localization of BrdU with GFAP labeling at this magnification. Moreover, in figure 3c they indicate that there is less colocalization of BrdU with GFAP in the sema4B-/- mice, suggesting that there is a selective decrease in astrocyte proliferation. However, as they point out, since the sema 4B-/- mice showed a specific decrease in GFAP expression, it is surprising that they didn't use a different astrocyte marker, such as S100ß, whose expression was unaffected by the absence of sema4B, for this analysis.”

To address the referees’ important comments and in order to circumvent the histology-based immunofluorescence pitfalls, we repeated the proliferation experiment as before (i.e., three injections of BrdU on days 3, 5 and 7), but instead of immunofluorescence, in this experiment we employed flow cytometry analysis. On day 7 (3 hours after the last BrdU injection), a biopsy of the injury site (3 by 3 mm of the cortex around the site of injury) was dissected out (after PBS perfusion to exclude cells in the blood) and the tissue dissociated into cells. The cells were then stained with anti-GLAST (astrocytes) and anti-BrdU (using qPCR and western blots we did not see a significant difference in the expression of GLAST between mutant and wild-type astrocytes). As seen in this new addition to Figure 3 (d and e), the results are similar to the immunostaining analysis, indicating that astrocytes in the Sema4B cortex are indeed less proliferative than wild-type astrocytes. Total proliferation at this time is also reduced. The results indicate that the reduced proliferation is mostly a reduction in astrocyte proliferation. However, since we have only tested relatively long time points (3-7 days), it is possible that other populations are affected at other time points. This point will be examined in a separate study.

3) “What is the in vitro injury indicated in figure 5c?”

In this experiment (fig. 5 and 6) a confluent monolayer of astrocytes was injured by passing a pipette tip (p200) through the middle of the plate. This explanation was added to the text.

4a) “The result with 4B S825D in figure 7j is unclear. Although the text indicates that both 4B-wt and S825D restored proliferation, the significance bar seems to indicate a difference between -/- with S825A and +/- control, not -/- with S825D.

Statistical analysis was added to the figure. We compared 4B S825D to Sema4B +/- astrocytes. The difference was not significant. Please note that although in Fig. 7j restoration of proliferation by the Sema4B SD mutant seems slightly lower then restoration of mutant astrocytes infected with Sema4B, this is not the case in Fig. 7k (astrocytes+/- infected with Sema4B or Sema4B-/- astrocytes infected with the mutant SD show the same level of proliferation (when stimulated with serum)). We are therefore confident that Sema4B SD is as capable of rescuing Sema4B activity as wt (we attribute this difference between 7j and 7k to variability between experiments).

4b) “Additionally, they conclude from their experiment in figure 7k that Sema 4B phosphorylation at serine 825 is required for serum-dependent astrocyte proliferation, but the experiment only shows that this phosphorylation can bypass the need for serum, not that it's necessarily required.”

We accept the referees’ comment and have changed the text accordingly.

5) “It is not clear from their method for growing astrocyte cultures how pure their preparation of astrocytes is. Although they state that 98-100% of their cells are S100ß-positive, they have not stained for the presence of contaminating cells, such as microglia and oligodendrocyte progenitors, which are likely to be present since their method is only to dissociate and plate the cells in serum-containing media with no shaking to remove other cell populations as is usually required to obtain a pure astrocyte culture.”

As suggested by the referees, we tested our astrocyte culture once more with S100b and the following antibodies: O4, Olig2, IbaI, and NeuN. We do not see contamination by other cell populations. We agree with the referees that the initial culture is likely contaminated with other cells. However, we split the initial culture and kept the cells with no serum for 3-4 days. This step reduced basal cell proliferation. After this period, we either tested cell proliferation or, in this case, stained to see the level of purity of our culture. In most experiments we saw only astrocytes. We believe that because of the split and/or the incubation without serum, we were left with a highly enriched population of astrocytes. It is also possible that due to our selection with D-valine during the initial astrocyte selection, other contaminating cells did not survive or became more sensitive to our serum starvation step.

6) “A general concern is that the phenotypes arise from developmental defects. The authors try to overcome this issue through the use of shRNAs and acute KD in vitro, but the results are puzzling and poorly controlled. There is a reduction of about 50-60% in the mRNA by the shRNAs while the heterozygous mice have no phenotypes. Importantly, protein reduction is not evaluated and the specificity of the shRNAs is not tested. Western blots of Sema4B are required in the KD experiments.”

To address this comment we tested the protein levels of Sema4B after KD (Fig. 4C). Protein samples from the original experiments were tested for the levels of Sema4B (we extracted protein during the experiments, but since we did not have a specific antibody for Sema4B at the time that these experiments were performed, the samples were not tested). As shown in Fig. 4C, Sema4B is almost not detected after KD at the protein level, suggesting that the effects of shRNA are much more efficient than those seen at the RNA level. We therefore conclude that both our KD and KO experiments consistently show the need for Sema4B for astrocyte proliferation.

From the results it seems that the effects of KD on astrocyte proliferation may have been more dramatic (Fig. 4d, about 60 and 70% reduction) than in the KO experiments (Fig. 4b, about 40% reduction). However, in a similar experiment carried out separately, there was a similar reduction of astrocyte proliferation as detected in the KD experiments (Fig. 7J, about 65% reduction). Although it is possible that KD has stronger effects on astrocyte proliferation, it is most likely that the differences only represent variability among experiments and do not represent a meaningful biological difference.

Regarding the possible developmental defects in the Sema4B mutant mice which might indirectly affect astrocyte proliferation, we would like to add that the experiments in which Sema4B mutant astrocytes were rescued with a Sema4B expression vector make this possibility unlikely.

7) “Following on from point 6 above, a rescue experiment of the shRNAs with an shRNA-resistant form of Sema4B is needed to verify specificity of the shRNA results.”

We agree that rescue experiments are very important when using shRNA or siRNA, especially if this is the only method used to repress gene expression. Technically, however, it is problematic in the case of astrocytes. Astrocytes are very difficult to infect with lentivirus and, in fact, we had to significantly concentrate the viruses (by ultracentrifugation) to even infect with just shRNA or Sema4B expression vectors. Unfortunately, we were not able to obtain viable astrocytes after selection with both blasticidin and puromycin (for the selection of both Lenti-Sema4B and Lenti-shRNA vectors). Nevertheless, since the shRNA is only complementary to our experiments with the Sema4B mutant astrocytes, and in general, the results are very similar, one can safely assume that Sema4B indeed has a function required for the proliferation of astrocytes.

8) “An optional addition to the data would be gene expression analysis of GFAP, Vimentin and LNC2, which were tested in vivo, in the non-phospho Sema4B infected astrocytes. This will indicate if the signaling from that site is contributing only to the proliferative response or also for astrogliosis associated gene expression.”

We agree with the referees that recapitulation of the modulatory role of Sema4B in astrocyte activation would have been a nice addition. We tried the suggested experiments; however, the variability among experiments with regard to the expression of GFAP and vimentin was too high and we did not see a consistent difference among experiments. LCN2 was slightly more consistent and showed higher expression in the knock-out astrocytes as compared to the control (like the results *in vivo*); however, the differences were small and the variability was high, and we thus did not include these results. Although the results were disappointing, it is important to note that in our *in vivo* system we are testing adult astrocytes, while *in vitro* we are testing newborn astrocytes (it is not possible to grow astrocytes from adult brain). The difference in the gene expression profile of p1 astrocytes as compared to adult brain astrocytes is quite significant, as reported previously (Cahoy *et al*., Journal of Neuroscience, 2008, 28(1): 264-278). Moreover, it is quite apparent that the culture process itself activates astrocytes and therefore complicates the analysis.
